# Electroacupuncture alleviates inflammatory pain by modulating the CXCL1/CXCR2 signaling axis in the spinal cord dorsal horn of rats

**DOI:** 10.3389/fnmol.2026.1880288

**Published:** 2026-09-11

**Authors:** Xiazhen Xu, Ziyan Fang, Kaihua Song, Yang Shen, Chenxin Jiang, Mengchao Xu, Shouhai Hong

**Affiliations:** 1 Department of Acupuncture-Moxibustion and Tuina, Zhejiang Chinese Medical University, Hangzhou, China; 2 Zhejiang Provincial Hospital of Chinese Medicine, Hangzhou, China

**Keywords:** analgesic, CXCL1, CXCR2, electroacupuncture, inflammatory pain, rat, spinal cord dorsal horn

## Abstract

**Aims:**

To elucidate the function of the C-X-C motif chemokine ligand 1 (CXCL1)-C-X-C Motif Chemokine Receptor 2 (CXCR2) signaling axis within the Spinal Cord Dorsal Horn (SCDH) in mediating the antinociceptive effects of electroacupuncture (EA) on pain.

**Main methods:**

An inflammatory pain model was created by injecting 0.1 ml of Complete Freund’s Adjuvant into the right foot of Wistar rats. EA was then applied daily for 7 days at the Zusanli and Kunlun points, using a 2/100 Hz wave pattern and gradually increasing the current from 0.5 mA to 1.5 mA. Pain thresholds were measured, and effective pain relief was confirmed. The spinal dorsal horn was analyzed for CXCL1 content via ELISA, CXCR2 expression via Western blotting, and gene expression via PCR. Intrathecal catheterization was used to administer recombinant CXCL1 or the CXCR2 antagonist SB225002 to study their effects on EA’s pain relief.

**Key findings:**

(1) Electroacupuncture demonstrates substantial analgesic effects in rats experiencing inflammatory pain, notably increasing the thermal and mechanical pain thresholds in the plantar region of the affected limb. (2) Findings from enzyme-linked immunosorbent assay (ELISA) and Western blot (WB) analyses reveal that, EA significantly downregulates the protein levels of CXCL1 and CXCR2 in the dorsal horn of the spinal cord in rats with inflammatory pain, and decreases the expression levels of the inflammatory cytokine IL-1β in serum.(3) PCR results indicated that EA significantly reduced CXCL1 and CXCR2 mRNA levels in the spinal cord’s dorsal horn, highlighting its gene regulatory effect. (4) Subsequent pretreatment with recombinant CXCL1 protein counteracted the analgesic effects of EA. In contrast, intrathecal administration of the CXCR2 antagonist SB225002 replicated these effects and led to a significant reduction in serum IL-1β levels. (5) Finally, the administration of recombinant CXCL1 and CXCR2 antagonists substantiated that CXCL1 and CXCR2 serve as pivotal regulatory targets for the analgesic effects of EA. The upregulation of CXCL1 diminishes these effects, whereas the antagonism of CXCR2 amplifies them.

**Significance:**

Electroacupuncture alleviates chronic inflammatory pain in rats by targeting the CXCL1-CXCR2 signaling axis in the SCDH, reducing CXCL1, CXCR2, and serum IL-1β levels, making it a potential therapeutic target.

## 1 Introduction

Surveys indicate that chronic pain affects up to half of the global population, with chronic inflammatory pain heavily impacting individuals and society (Kang et al., 2023; Sommer and Rittner, 2024). Pain management remains a major challenge in medicine (Rometsch et al., 2025). Opioids, the standard for severe pain treatment, are limited by side effects like constipation, apnoea, tolerance, addiction, and overdose deaths. NSAIDs, used for inflammatory pain (Mattson et al., 2021; Zhuang et al., 2022), also pose risks such as gastrointestinal bleeding and cardiovascular issues (Gahbauer et al., 2023). Thus, there is an urgent need for new pain relief methods with fewer side effects or non-drug treatments (Király et al., 2018).

Acupuncture, a 3000-year-old Asian medical practice, was acknowledged by the WHO in 2003 for treating various diseases (Guo and Lu, 2024). Its global popularity is rising as a non-drug therapy due to its antinociceptive and pain-relief benefits, minimal side effects, and affordability, especially during the opioid crisis (Nielsen et al., 2022; Niemtzow, 2018). Although safe and effective for inflammatory pain, its mechanisms require more research (Zhang et al., 2023).

During inflammation, glial cells like astrocytes and microglia release pain-enhancing substances, including proinflammatory cytokines such as TNF-α and chemokines such as CXCL1 (Ji et al., 2013). Chemokines, small cytokines binding to GPCRs, activate signaling pathways and attract immune cells through chemotaxis (Wang et al., 2022). They are divided into four subfamilies: CXC, CC, C, and CX3C (Wang et al., 2018). The CXC-type chemokines include CXCL1–CXCL16, which feature a highly conserved motif consisting of two cysteines separated by any amino acid (Jiang et al., 2023). During the inflammatory process, CXC chemokines draw immune cells like neutrophils and lymphocytes to inflammation sites, activating receptors like CXCR1 and CXCR2, which enhance inflammation and pain (Le et al., 2004). Specifically, chemokines CXCL1–8 are known to attract neutrophils through chemotactic mechanisms (Rajarathnam et al., 2019), whereas CXCL9–11 are involved in the recruitment of activated T cells (Ullah et al., 2023).

C-X-C motif chemokine ligand 1, a member of the CXC chemokine family, is also referred to as growth-regulating oncogene α/keratinocyte-derived chemokine (GRO/KC) or cytokine-induced neutrophil chemotactic factor-1 (CINCL). It is a well-established neutrophil chemotactic factor (Korbecki et al., 2022) and plays a significant role in promoting both nociceptor and central sensitization through its primary receptor, CXCR2. Consequently, CXCR2 is considered a promising target for the development of novel analgesic drugs (Silva et al., 2017). The CXCL1-CXCR2 signaling pathway plays a crucial role in the modulation of pain sensitivity across various pain models (Jiang et al., 2023).

Empirical evidence indicates that astrocytes in the spinal cord produce CXCL1, which subsequently interacts with CXCR2 receptors on neurons, leading to the phosphorylation of ERK. This cascade promotes the expression of c-fos, cyclooxygenase-2, and NK-1, ultimately contributing to the development of chronic pain (Jiang et al., 2023). In the context of pathological pain, CXCL1 and CXCR2 directly influence injury sensors, enhancing neuronal excitation by modulating sodium channels NaV1.1 and NaV1.7, thereby perpetuating pain (Zhang et al., 2013). Additionally, CXCL1 secreted by astrocytes engages with CXCR2 on neurons, facilitating both neuropathic and inflammatory pain (Gao et al., 2024; Moraes et al., 2020). These findings suggest that the CXCL1-CXCR2 signaling axis represents a promising therapeutic target for inflammatory pain. Our previous research has demonstrated that EA, while alleviating inflammatory pain, significantly increases CXCL1 protein levels in the bloodstream. This elevated CXCL1 is implicated in mediating EA’s analgesic effects on inflammatory pain (Hong et al., 2021). The involvement of the CXCL1-CXCR2 signaling axis within the dorsal horn of the spinal cord in mediating the antinociceptive effects of EA remains insufficiently understood, with the underlying mechanisms yet to be fully elucidated.

This study aims to explore the role of the CXCL1/CXCR2 signaling pathway in the SCDH in the context of electroacupuncture-induced analgesia. By doing so, it seeks to contribute additional scientific evidence regarding the mechanistic action of EA in the mitigation of inflammatory pain.

## 2 Materials and methods

### 2.1 Experimental animals

Animals: Following the Laboratory Animal Guide (GB/T35892-2018), the study protocol was approved (License Number: IACUC-20220829-01). Adult SPF-grade Wistar male rats (250 ± 20 g) were purchased from Shanghai Slack Laboratory Animal Co., Ltd., via the Laboratory Animal Center of Zhejiang Chinese Medical University (Zhejiang, China), license number: SYXK (Hu) 2022-0012. All animals were fed in cages with free access to water, naturally illuminated according to circadian rhythm, room temperature 20 °C–26 °C, and relative humidity 50%–70%.

Anesthesia and euthanasia: For the purpose of intrathecal catheterization, rats were anesthetized using isoflurane inhalation, with concentrations of 3%–5% for induction and 1%–2% for maintenance. Prior to sample collection, the rats were subjected to deep anesthesia through an intraperitoneal (i.p.) injection of pentobarbital sodium at a dosage of 50 mg/kg. Euthanasia was conducted under deep anesthesia by means of transcardiac perfusion with ice-cold sterile normal saline. In brief, the thoracic cavity was opened, and a perfusion needle was inserted into the left ventricle to administer saline until the liver appeared pale and the effluent was clear. All animal procedures adhered to the ARRIVE guidelines and were conducted in compliance with the institutional animal care protocols approved by Zhejiang Chinese Medical University (IACUC-20220829-01).

### 2.2 Establishment of a rat model of inflammatory pain

The development of a rat model for inflammatory pain was achieved using Complete Freund’s Adjuvant (CFA), which comprises heat-inactivated, desiccated Mycobacterium tuberculosis H37Ra, liquid paraffin, and mannitol monooleate (Salam et al., 2025; Xu et al., 2018). We selected Complete Freund’s Adjuvant (CFA) due to its well-documented application in inducing inflammatory pain (Defaye et al., 2024; Marty-Lombardi et al., 2024).

**FIGURE 1:**
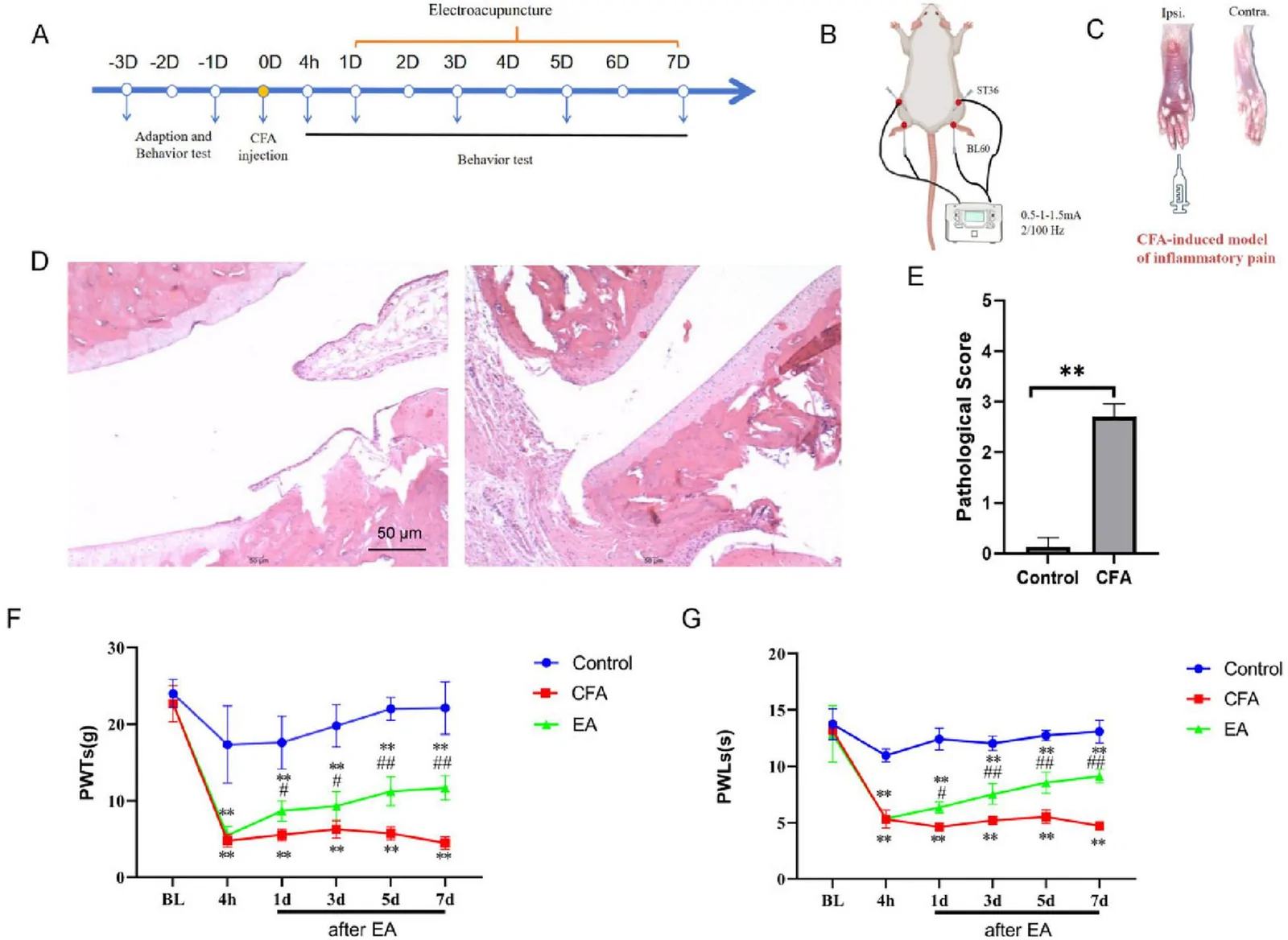
EA can alleviate mechanical and thermal pain in rats. **(A)** Schematic representation of the experimental timeline. **(B)** CFA-induced model of inflammatory pain. **(C)** Schematic Diagram of EA at Bilateral ST36 (5 mm laterally of the anterior tibial tubercle) and BL60 (at the level of the ankle joint, between the lateral malleolus and the calcaneal tubercle) in a rat. **(D)** Photos and H&E-stained sections show that, on day 7 after plantar s.c. injection of CFA in rats, the control group (left) displayed no discernible inflammatory lesions, whereas the CFA group (right) showed the synovial lining layer showed marked hyperplasia, with some cells exhibiting edema and inflammatory cell infiltration. Scale bar = 50 μ m. **(E)** Summary of histopathological scores of H&E staining (unpaired *t*-test, *n* = 6). **(F)** Effects of EA on mechanical pain thresholds. *p*-values indicate the difference in the threshold between Control vs. CFA; Control vs. EA; CFA vs. EA; (*n* = 6, two-way ANOVA). **(G)** Effects of EA on thermal pain thresholds. *p*-values indicate the difference in the threshold between Control vs. CFA; Control vs. EA; CFA vs. EA; (*n* = 6, two-way ANOVA). **P < 0.01 vs. control group. ^#^*P* < 0.05 vs. CFA. ^##^*P* < 0.01 vs. CFA. Data are represented as mean ± SEM; SEM, standard error of mean; EA, electroacupuncture.

Rats assigned to the model and EA groups received an intradermal injection of 0.1 ml CFA (1 mg/ml, Sigma Corporation, USA) into the right plantar surface, whereas those in the control group were administered an equivalent volume of normal saline. The rat model was deemed successfully established when the subjects exhibited signs of skin redness, swelling, and behaviors such as foot licking, limping, and avoidance of ambulation on the affected side post-injection (Figure 1C). Seven days following CFA administration, ankle joints were harvested for histopathological analysis to confirm the successful establishment of the model.

### 2.3 Nocifensive behavior assay

Mechanical allodynia: Prior to the baseline assessment, adaptive pain measurement was conducted over a period of three consecutive days. During this period, the rats were acclimated to the environment by being placed in Plexiglas transparent observation chambers on an elevated wire mesh for a duration of 30 min. A series of von Frey hairs (UGO Basile, Italy), with forces of 0.6, 1, 1.4, 2, 4, 6, 8, 15, and 26 grams, were employed to perpendicularly stimulate the posterior plantar region of the rats, following previously established methodologies (Ren et al., 2024). Each filament was bent into an “S” shape and applied for 3 to 5 s to observe any abrupt paw withdrawal, licking, or shaking responses, which were interpreted as indicative of pain-like behavior. An interval of at least one minute was maintained between successive stimulations to prevent any carryover effects from prior stimulations. The pain threshold was determined using the “up-down” testing paradigm (Li et al., 2025), and the 50% paw withdrawal threshold (PWT) was calculated using a nonparametric Dixon test (Chaplan et al., 1994; Dixon, 1980). All assessments were performed by an experimenter who was blinded to the experimental conditions.

Heat hyperalgesia: The Hargreaves test was employed for this assay. Following an acclimation period, a radiant light beam, generated by a focused light source, was directed at the plantar surface of the rat’s hind paw to measure paw withdrawal latency (PWL) using the Hargreaves Plantar Test Apparatus (Ugo Basile, Italy). Upon the rat experiencing pain and withdrawing its paw, the light source was automatically extinguished, and the timer ceased operation. The PWL was subsequently determined and recorded. A cutoff threshold of 20 s was established to prevent excessive heating that could result in tissue injury. All tests were conducted by an experimenter blinded to the experimental conditions.

### 2.4 Electroacupuncture (EA) intervention

Electroacupuncture was administered by a qualified acupuncturist in accordance with the T/CAAM0002–2020 Nomenclature and Location of EA Points for Laboratory Animals Part 2: Rat (China Association of Acupuncture and Moxibustion, 2025). The bilateral Zusanli (ST36) and Kunlun (BL60) acupoints were selected (Wang et al., 2020). The ST36 acupoint is situated posterolateral to the knee joint, approximately 5 mm below the capitulum fibulae, while the BL60 acupoint is located in the depression between the lateral malleolus and the Achilles tendon of the hindlimb. In the EA group, the rats were gently restrained using soft cloth to facilitate smooth needle insertion. In the EA group, treatment began 24 h post-modeling. Needles, measuring 0.18 mm in diameter and 13 mm in length, were inserted to a depth of 3–5 mm at the bilateral “Zusanli” (ST36) and “Kunlun” (BL60) acupoints (Oh and Kim, 2022; Wang et al., 2020). These needles were connected to a Hans acupoint nerve stimulator (Hans-200A, Huawei Co., Ltd., Beijing, China), with parameters set at 2/100 Hz for square-wave current output (pulse width: 0.2 ms), and a current intensity ranging from 0.5 to 1.5 mA, increased by 0.5 mA increments every 10 min, for a total duration of 30 min (Figure 1B) (Fang et al., 2021; Shi et al., 2020; Wu et al., 2025; Xiang X. et al., 2019). In the sham EA group, needles were inserted subcutaneously without electrical stimulation. Both EA and sham EA treatments commenced 24 h post-modeling and were administered once daily for seven consecutive days (Wang et al., 2021; Xu et al., 2022).

### 2.5 Preparation of spinal cord dorsal horn samples

Following the final behavioral assessment on day 7, the rats were subjected to deep anesthesia using pentobarbital(50 mg/kg) administered via intraperitoneal injection. Subsequently, transcardiac perfusion was conducted utilizing ice-cold sterile normal saline to effectively remove blood from the systemic circulation. The spinal column was then swiftly dissected to expose the lumbar enlargement, specifically the L4–L6 segments. The spinal cord was meticulously flushed out with sterile normal saline using a 50 mL syringe. The dorsal horn was carefully isolated on ice, promptly frozen in liquid nitrogen, and stored at −80 °C for subsequent analysis.

### 2.6 Western blotting

On the seventh day following modeling, pain threshold testing was conducted. Subsequently, the dorsal horn of the spinal cord (SCDH) from segments 4–6 on the ipsilateral side was harvested on ice post-perfusion and promptly stored at −80 °C. The collected tissue was then placed in RIPA lysis buffer (Biyuntian, Cat. No. P0013B) supplemented with protease inhibitors (RIPA lysis buffer: PMSF = 50:1) and homogenized using a pre-chilled high-speed, low-temperature homogenizer (operated for 30 s, paused for 15 s, repeated four times, at a frequency of 60). The homogenized samples were subsequently centrifuged at 4 °C and 12,000 rpm for 15 min, and the supernatant was collected. Protein concentration was determined using the BCA protein concentration assay kit (Biyuntian, Cat. No. P0010S). A total of 20 μg of protein was loaded into each lane. The target protein was separated on an 8% or 15% gel and subsequently transferred to a polyvinylidene fluoride (PVDF) membrane utilizing a rapid semi-dry protein transfer system (Bio-Rad, USA). The membranes were initially blocked using 5% bovine serum albumin (BSA) at ambient temperature for a duration of 1 h. Subsequently, the primary antibody (CXCR2, 1:1000; GTX14935, GENETEX, USA) was introduced, and the samples were incubated overnight at 4 °C with gentle agitation. Following this, the appropriate secondary antibody was applied, and the samples were further incubated at room temperature for 1 h. A mouse anti-GAPDH antibody (1:10000; ab8245, Abcam, UK) served as an internal control. Immunoreactivity was detected utilizing an extra-ultrasensitive enhanced chemiluminescent (ECL) substrate (BioSharp, China) and visualized with the FluorChem R system (Biotechne, USA). The gray values of each band were quantitatively analyzed using ImageJ software [National Institutes of Health (NIH)].

### 2.7 ELISA for CXCL1 and IL-1β

On the seventh day following EA or sham EA treatment, rats were euthanized. Ipsilateral L4-6 spinal cord dorsal horn (SCDH) tissues and serum samples were collected and immediately preserved in liquid nitrogen. The enzyme-linked immunosorbent assay (ELISA) protocol was adapted from previous studies with slight modifications (Hong et al., 2021; Liu et al., 2016; Yin et al., 2024). Tissue homogenization was conducted in a solution of 50 mM Tris base (pH 7.4) and 150 mM sodium chloride, supplemented with protease inhibitor (PMSF, Beyotime, China) and 0.2% Triton X, using a high-speed cryogenic grinder (Servicebio, China). The homogenization process was carried out at maximum speed for 20 min. Subsequently, the samples underwent centrifugation at 10,000 × *g* for 10 min at 4 °C. The supernatant was collected for analysis, and CXCL1 in SCDH tissues (R&D Systems, USA) and IL-1β in serum (Nanjing Jiancheng, China) were quantified according to the manufacturers protocols. Absorbance measurements were taken at 450 nm using a microplate reader (Thermo Fisher Varioskan LUX, USA), with wavelength correction at 540 nm. The total protein concentration was determined using the BCA assay (Beyotime, China).

### 2.8 Histopathological assessment of ankle joint

The pathological examination of the ipsilateral ankle joints was performed subsequent to the behavioral assessments conducted on day seven (Inglis et al., 2005). The tissues were post-fixed in 10% neutral buffered formalin for a duration of 24 h, and subsequently decalcified using 10% nitric acid. The decalcified tissues were then embedded in paraffin blocks, sectioned longitudinally at a thickness of 4 μm, and stained with hematoxylin and eosin (H&E) for morphological examination under a light microscope at magnifications of ×10 and ×40. The pathological scoring system was defined as follows: a score of 0 indicated a normal ankle joint; a score of 1 denoted normal synovium with occasional immune cells; a score of 2 represented definite arthritis characterized by a few layers of flat to rounded synovial lining cells, scattered mononuclear cells, and foreign body granuloma formation, or dense infiltration with immune cells and foreign body granuloma formation; a score of 3 indicated clear hyperplasia of the synovium with three or more layers of loosely arranged lining cells, dense infiltration of immune cells, and foreign body granuloma formation; and a score of 4 corresponded to severe synovitis, foreign body granuloma formation, and potential erosion of the articular cartilage and subchondral bone (Xu et al., 2018). The analysis was conducted in a blinded manner using ×40 objectives. Three sections were obtained from each sample, and the results were averaged to derive the final outcome.

### 2.9 Determination of Cxcl1 and Cxcr2 RNA levels in the SCDH by quantitative polymerase chain reaction

The ipsilateral dorsal horn of the spinal cord segments L4–L6 was excised and promptly preserved in TRIzol solution (Thermo Fisher Scientific, USA). Total RNA extraction was conducted utilizing TRIzol reagent supplemented with deoxyribonuclease I (DNase I) to eliminate any contaminating DNA. The purity and concentration of the samples were evaluated using a NanoDrop spectrophotometer (NanoDrop Products, USA). Quantitative PCR (qPCR) was executed employing the LightCycler 480 SYBR Green I Master mix (Roche, Switzerland) within a 20 μl reaction volume, following the manufacturer’s protocol, on a LightCycler 480 Real-Time PCR System (Roche, Switzerland). Each reaction was conducted in triplicate and normalized to the expression of the β-actin (Actb) gene. The cycle threshold (CT) values for each well were determined using the LightCycler 480 system software, and the mean value of the three replicates was calculated. Relative quantification was performed using the ΔΔCT method, as described in references (Nie et al., 2025). Detailed primer sequences are provided in Supplementary Table 1. The Ct values of β-actin are provided in Supplementary Table 2.

### 2.10 Intrathecal injection and drug administration

#### 2.10.1 Preparation of recombinant CXCL1 protein

A stock solution of recombinant CXCL1 protein should be prepared at a concentration of 100 μg/mL in sterile phosphate-buffered saline (PBS) and stored at −20 °C. Thirty minutes prior to administration, the high-dose CXCL1 solution should be diluted from the stock solution using sterile PBS to achieve a final concentration of 10 ng/μL, while the low-dose CXCL1 solution should be diluted to a concentration of 1 ng/μL.

#### 2.10.2 Preparation of CXCR2 antagonist

Dissolve 5 mg of SB225002 in dimethyl sulfoxide (DMSO) to create a stock solution with a concentration of 20 mg/mL, and store it at −20 °C. Thirty minutes before administration, a portion of the high-dose SB225002 stock solution should be diluted with PBS to a concentration of 2 μg/μL, and the low-dose SB225002 solution should be diluted to a concentration of 1 μg/μL.

### 2.11 Drug administration and intrathecal injection

Animals were randomly divided into the following three parts (*n* = 6 in each group of Experiment 1; *n* = 5 in each group of Experiment 2 Part 1; *n* = 5 in each group of Experiment 2 Part 2): control, CFA, CFA++EA, CFA+PBS, EA+PBS, EA+low-dose recombinant CXCL1, EA+high-dose recombinant CXCL1, CFA+10% DMSO, EA+10% DMSO, CFA+SB225002+10% DMSO (CXCR2 antagonist). The recombinant CXCL1 protein and SB225002 were procured from R&D Systems and Sigma, respectively, with catalog numbers 515-CN-050/CF and SML0716-5MG. Intrathecal administration was conducted using a 10 μl micro-syringe.

Following anesthesia with isoflurane inhalation anesthesia (3%–5% for induction, 1%–2% for maintenance) via a veterinary anesthesia machine, an incision was made in the skin at the most prominent spinous process of the iliac crest, the muscles were carefully dissected, and the spinous process was excised. A 22 cm PE10 catheter filled with saline was inserted at an angle of 30–45° until the rat exhibited tail-wagging and leg-twitching, which indicated accurate placement. The catheter was then secured and routed subcutaneously to emerge at the back of the neck, with the catheter tip being heated to prevent slippage. The muscles and skin were sutured in layers, and the site was disinfected with penicillin. Intrathecal injection of lidocaine was employed to verify catheter placement; any cases resulting in paralysis of both hind limbs were considered placement failures and subsequently excluded from the study.

Drug administration was conducted 30 min prior to EA treatment, involving the intrathecal injection of 10 μl of recombinant CXCL1 protein (at either low or higher dose) or SB225002, followed by 25 μl of saline to ensure complete delivery of the drug into the spinal cord without residue. The control group received an equivalent volume of PBS or 10 μl of 10% dimethyl sulfoxide (DMSO). Daily EA treatment commenced 24 h following model induction and continued for 7 consecutive days. The mechanical and thermal pain thresholds of the rats were assessed at predetermined time points.

Animals exhibiting evident motor dysfunction following intrathecal procedures, experiencing unsuccessful drug administration, or encountering unforeseen mortality during the experimental period were excluded from data analysis. The final count of biological replicates for each test is specified in the respective figure legends.

### 2.12 Statistical analysis

Statistical analyses were conducted utilizing SPSS software, version 26.0 (IBM, New York, USA). Experimental data are presented as means ± SEM, with sample sizes ranging from 4 to 10. Data visualization was performed using GraphPad software (GraphPad, San Diego, CA, USA). Analysis of body weight data was carried out using repeated measures ANOVA via GraphPad. Comparisons among multiple groups were executed using one-way ANOVA, followed by post-hoc analyses employing either LSD or Tamhane’s T2 tests. Both independent samples *t*-test and Pearson correlation analysis were used to analyze intergroup differences and linear associations separately. *P* < 0.05 was considered to indicate statistical significance.

## 3 Results

### 3.1 Electroacupuncture (EA) intervention ameliorates mechanical and thermal pain hypersensitivities in a rat model of inflammatory pain induced by complete Freund’s adjuvant

Following the establishment of the CFA-induced inflammatory pain model, baseline data were obtained from rats in each experimental group prior to the induction of the model. The mechanical pain thresholds (PWT) were evaluated using the von Frey filament method, while the thermal pain thresholds (PWL) were assessed via the plantar heat radiation method. These measurements were recorded 4 h post-modeling, as well as on days 1, 3, 5, and 7 subsequent to EA treatment (Figure 1A). Statistical analysis revealed no significant differences in baseline mechanical pain thresholds (Figure 1F) among the three groups of rats (*P* > 0.05). In contrast to the sham-model group, the mechanical pain threshold in the model group was significantly reduced 4 h post-modeling (*P* < 0.01), and this reduction persisted until day 7, thereby confirming the successful establishment of the model. To validate the establishment of the CFA-induced inflammatory model at the histological level, we performed histopathological staining on the ankle joint 7 days following CFA injection. In the control group, the synovial tissue was observed to be smooth and transparent, lacking any signs of hyperemia or hypertrophy; the synovial cells were clearly defined, showing no signs of enlargement or deformation, and there was an absence of significant inflammatory cell infiltration. In contrast, the model group exhibited marked hyperplasia of the synovial lining layer, with some cells showing edema and evidence of inflammatory cell infiltration. This was reflected in a significantly elevated histopathological score in the CFA group (*P* < 0.01, Figures 1D, E).

In the EA cohort, the mechanical pain threshold was notably diminished 4 h post-modeling (*P* < 0.01). Relative to the model group, EA administered at the bilateral Zusanli (ST36) and Kunlun (BL60) acupoints resulted in a significant enhancement of the mechanical pain threshold from day 1 (*P* < 0.05), with a more pronounced increase observed between days 3 and 7 (*P* < 0.01). Following this, thermal pain thresholds were assessed in the rats (Figure 1G). Initial thermal pain thresholds did not differ significantly among the three groups (*P* > 0.05), indicating that the groups were well-balanced and comparable. Both the model and EA groups demonstrated a highly significant reduction in thermal pain thresholds 4 h post-modeling compared to the sham-model group (*P* < 0.01), thereby validating the effectiveness of the modeling procedure. Compared to the model group, the EA group exhibited a significant increase in thermal pain thresholds on day 1 (*P* < 0.05). In summary, EA demonstrates substantial analgesic properties and effectively ameliorates thermal hyperalgesia in rats experiencing inflammatory pain.

### 3.2 Electroacupuncture reduced the expression of CXCL1 and CXCR2 proteins in the dorsal horn of the spinal cord dorsal horn (SCDH) in rats with inflammatory pain

**FIGURE 2:**
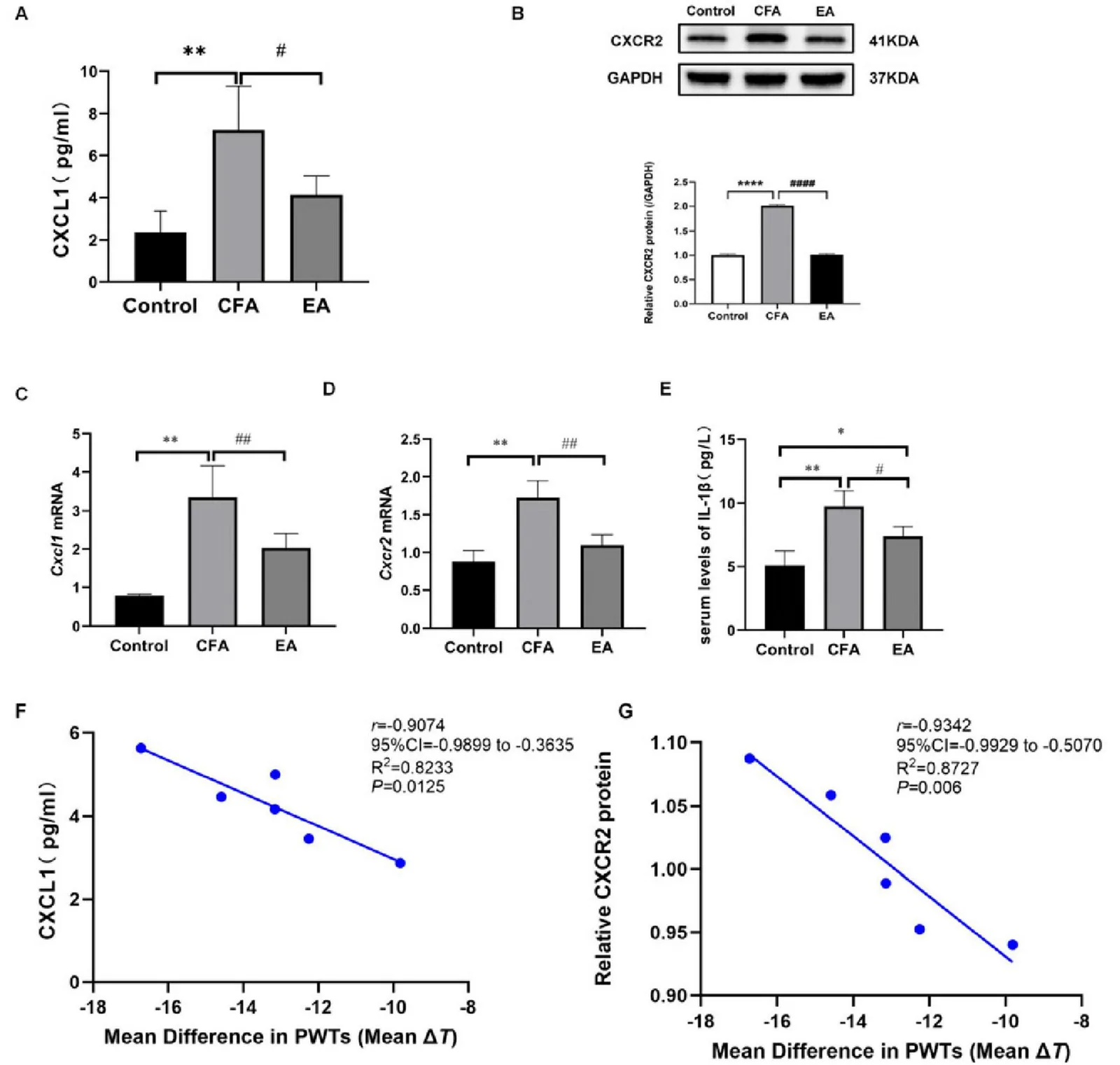
EA can decline the expression of CXCL1 and CXCR2 mRNA and protein in the SCDH, and it can downregulate the levels of the pro-inflammatory cytokine IL-1β in the serum of rats with inflammatory pain. **(A)** Protein expression of CXCL1 (*n* = 6, one-way ANOVA). **(B)** Protein Expression of CXCR2 (*n* = 6, one-way ANOVA). **(C)** Expression of mRNA in CXCL1 (*n* = 6, one-way ANOVA). **(D)** Expression of mRNA in CXCR2 (*n* = 6, one-way ANOVA). **(E)** Enzyme-linked immunosorbent assay (ELISA) determination of serum levels of IL-1β (*n* = 6, one-way ANOVA). **(F,G)** Linear correlations between EA analgesic magnitude (Mean Δ*T* of PWTs) and spinal dorsal horn CXCL1 (left)/CXCR2 (right) protein levels in individual rats (*n* = 6; Pearson correlation analysis). All data are presented as the means ± SEM; *P < 0.05; **P < 0.01; ****P < 0.0001 vs. control; ^#^*P* < 0.05; ^##^*P* < 0.01; ^####^ *P* < 0.0001 vs. CFA.

To investigate whether EA mediates its analgesic effects through the modulation of CXCL1 and CXCR2 proteins in the dorsal horn of the spinal cord (SCDH), we initially quantified CXCL1 protein expression utilizing an enzyme-linked immunosorbent assay (ELISA) as shown in Figure 2A. Our findings indicated a significant upregulation of CXCL1 protein expression in the SCDH of the experimental group compared to the sham-treated group (*P* < 0.01). Importantly, a 7-day EA intervention at the bilateral ST36 and BL60 acupoints effectively reversed this upregulation, resulting in a significant downregulation of CXCL1 protein expression relative to the experimental group (*P* < 0.05). To substantiate the regulatory impact of EA on the CXCL1-CXCR2 signaling pathway, we subsequently assessed CXCR2 protein expression via Western blot (WB) analysis. Results demonstrated (Figure 2B) a substantial increase in CXCR2 protein expression in the model group compared to the sham-model group (*P* < 0.0001). Whereas the 7-day course of EA treatment remarkably significantly attenuated the elevated CXCR2 protein levels in comparison to the model group (*P* < 0.0001). The findings of this study demonstrate that inflammatory pain leads to the upregulation of CXCL1 and CXCR2 proteins in the dorsal horn of the rat spinal cord. Conversely, EA has been shown to mitigate this aberrant protein overexpression in the dorsal horn.

### 3.3 Electroacupuncture decreased the mRNA levels of CXCL1 and CXCR2 in the spinal cord dorsal horn (SCDH) in rats with inflammatory pain

To further investigate the regulatory impact of EA on the gene transcription of the CXCL1-CXCR2 signaling axis within the spinal cord dorsal horn (SCDH) of rats experiencing inflammatory pain, we employed quantitative real-time polymerase chain reaction (qRT-PCR) to assess the mRNA expression levels of Cxcl1 and Cxcr2. The findings indicated that (Figure 2C), relative to the sham-model group, the mRNA expression of Cxcl1 in the SCDH was significantly upregulated in the model group (*P* < 0.01). Following 7 consecutive days of EA treatment, the elevated Cxcl1 mRNA levels in the EA group were significantly downregulated in comparison to the model group (*P* < 0.01). Similarly (Figure 2D), the mRNA expression of Cxcr2 in the SCDH was markedly increased in the model group compared to the sham-model group (*P* < 0.01). A 7-day EA intervention effectively reversed the inflammatory pain-induced upregulation of Cxcr2 mRNA expression, resulting in a highly significant reduction in Cxcr2 mRNA levels relative to the model group (*P* < 0.01). The aforementioned results demonstrated that the transcription of CXCL1-CXCR2 signaling was activated in rats following the successful induction of inflammatory pain, and that EA treatment significantly suppressed the excessive mRNA expression of Cxcl1 and Cxcr2.

To investigate the individual correlation between EA-induced analgesia and the expression of CXCL1/CXCR2, we conducted a Pearson correlation analysis (Figures 2F–G). Pearson correlation tests were specifically performed between Mean Δ*T* and spinal CXCL1/CXCR2 expression. The results revealed significant strong negative correlations between Mean Δ*T* and CXCL1 (*r* = −0.9074, *P* = 0.0125), as well as between Mean Δ*T* and CXCR2 (*r* = −0.9342, *P* = 0.006). Rats exhibiting the most pronounced increase in pain threshold following electroacupuncture intervention demonstrated the lowest levels of CXCL1 and CXCR2 protein expression in the spinal cord.

### 3.4 Electroacupuncture can downgrade the levels of IL-1β in the serum of rats with inflammatory pain.

Interleukin-1 beta (IL-1β), a key pro-inflammatory cytokine, plays a pivotal role in the initiation and progression of inflammatory pain by mediating and sustaining inflammatory responses and inducing hyperalgesia (Si et al., 2024). To further elucidate the antinociceptive mechanisms underlying the analgesic effects of EA, serum levels of IL-1β were quantified in rats from each experimental group using the enzyme-linked immunosorbent assay (ELISA) technique. The ELISA results indicated that (Figure 2E), in comparison to the sham model group, CFA-induced inflammatory pain significantly increased serum IL-1β levels in rats (*P* < 0.01). Following a 7-day regimen of EA treatment, the serum levels of interleukin-1 beta (IL-1β) in the EA cohort were markedly reduced compared to those observed in the model group (*P* < 0.05). This finding suggests that EA is effective in diminishing the serum concentration of the pro-inflammatory cytokine IL-1β in rats experiencing inflammatory pain.

### 3.5 Intrathecal injection of recombinant CXCL1 can reverse the antinociceptive effects of electroacupuncture, and there is a time-dependent and dose-dependent relationship; it has no effect on serum IL-1β levels

**FIGURE 3:**
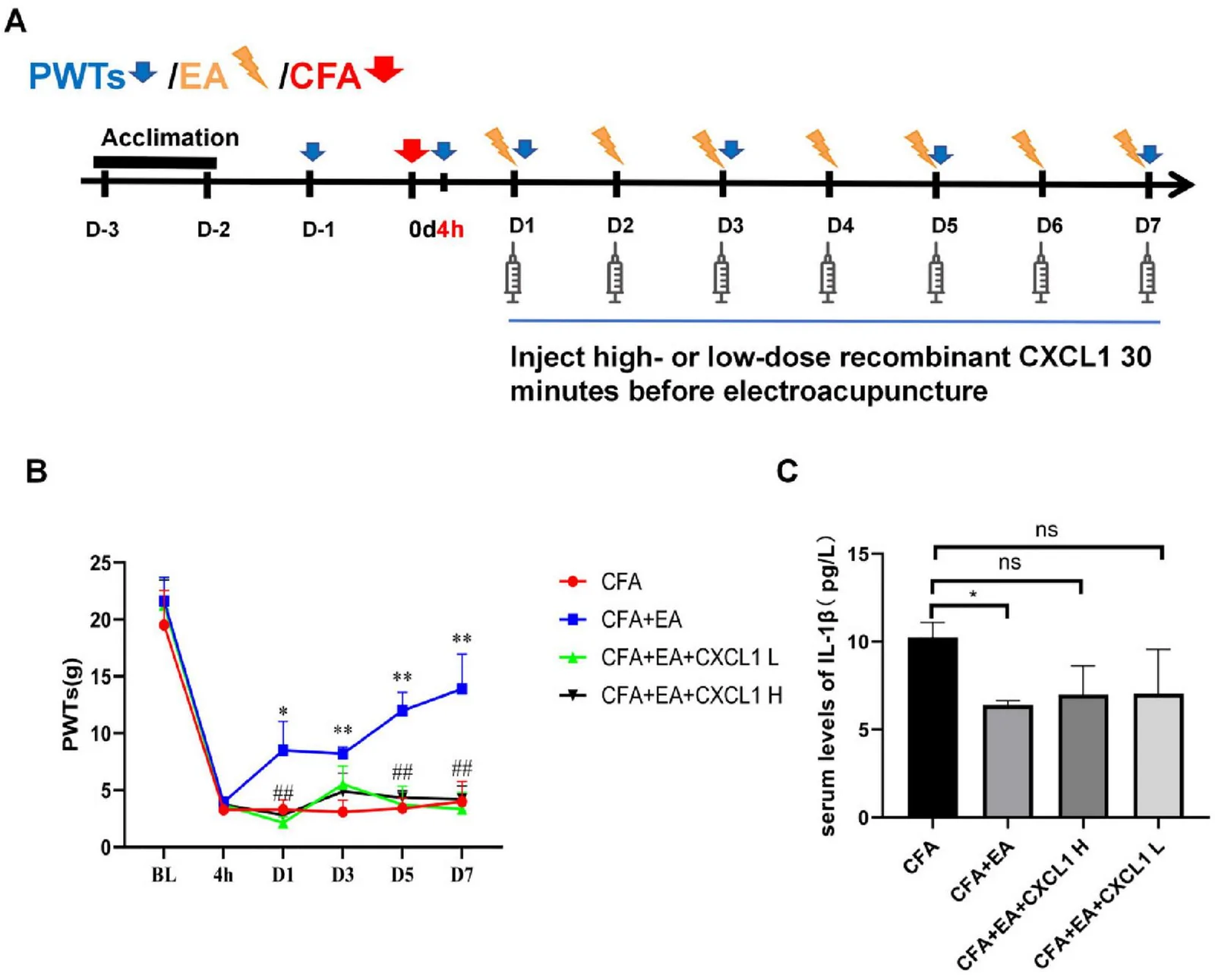
Intrathecal injection of recombinant CXCL1 reverses the analgesic effects of EA. **(A)** Schematic representation of the experimental timeline. **(B)** The Effect of Upregulating the Medial CXCL1 in the SCDH on electrostimulation-induced inflammatory pain and mechanical pain in rats (*n* = 4, two-way ANOVA). **(C)** The effect of increased CXCL1 in the SCDH on serum IL-1β levels in rats with EA-induced inflammatory pain (*n* = 4, one-way ANOVA). *P < 0.05 vs. CFA;**P < 0.01 vs. CFA. ^##^*P* < 0.01 vs. CFA+EA; Data were analyzed with a two-way repeated measures ANOVA followed by *post hoc* Bonferroni tests. The results are expressed as the means ± SEM; SEM, standard error of mean; EA, electroacupuncture; The CFA+EA+CXCL1L group received an intrathecal injection of 1 ng/μL of recombinant CXCL1; the CFA+EA+CXCL1H group received an intrathecal injection of 10 ng/μL of recombinant CXCL1; the CFA group and the CFA+EA group received intrathecal injections of 10 μL of PBS solution (equivalent volume).

In the subsequent phase of our study, we aim to examine the role of CXCL1 in mitigating inflammatory pain through the application of EA. To achieve this, we employed intrathecal injection to artificially elevate the concentration of CXCL1 in the SCDH and evaluated its impact on the antinociceptive effects of EA (Moraes et al., 2020). Recombinant CXCL1 protein was administered via intrathecal injection 30 min prior to the EA intervention (Hong et al., 2021). Mechanical pain thresholds (PWT) and thermal pain thresholds (PWL) were measured in rats using the von Frey filament method and the plantar heat radiation method at intervals of 4 h, 1 day, 3 days, 5 days, and 7 days post-modeling. The detailed procedure is illustrated in Figure 3A. The findings indicated that Figure 3B, in comparison to the model group, the EA group exhibited a significant increase in mechanical pain threshold on the first day following EA treatment (*P* < 0.05). In comparison to the EA group, the low-dose group receiving a combination of EA and recombinant CXCL1 exhibited a highly significant reduction in the mechanical pain threshold on the first day post-administration (*P* < 0.01). No statistically significant difference was observed on day 3; however, a highly significant decrease in the mechanical pain threshold was noted again on days 5 and 7 (*P* < 0.01). In the group receiving EA in conjunction with high-dose recombinant CXCL1, a significant reduction in the mechanical pain threshold was observed in rats (*P* < 0.01) following the first day of treatment. This reduction persisted until the seventh day, suggesting that the intrathecal administration of recombinant CXCL1 counteracted the analgesic effects of EA and there is a time-dependent and dose-dependent relationship.

Additionally, ELISA analyses (Figure 3C) indicated that the intrathecal injection of recombinant CXCL1 did not significantly alter serum IL-1β levels across all groups (*P* > 0.05). This finding implies that CXCL1 primarily mitigates the antinociceptive effects of EA by modulating local signaling pathways within the spinal cord.

### 3.6 Antagonizing the CXCR2 receptor can up-regulate the mechanical pain threshold of rats with inflammatory pain, which is similar to the analgesic effects of electroacupuncture. it can also down-regulate the content of IL-1β in the serum of rats with inflammatory pain

**FIGURE 4:**
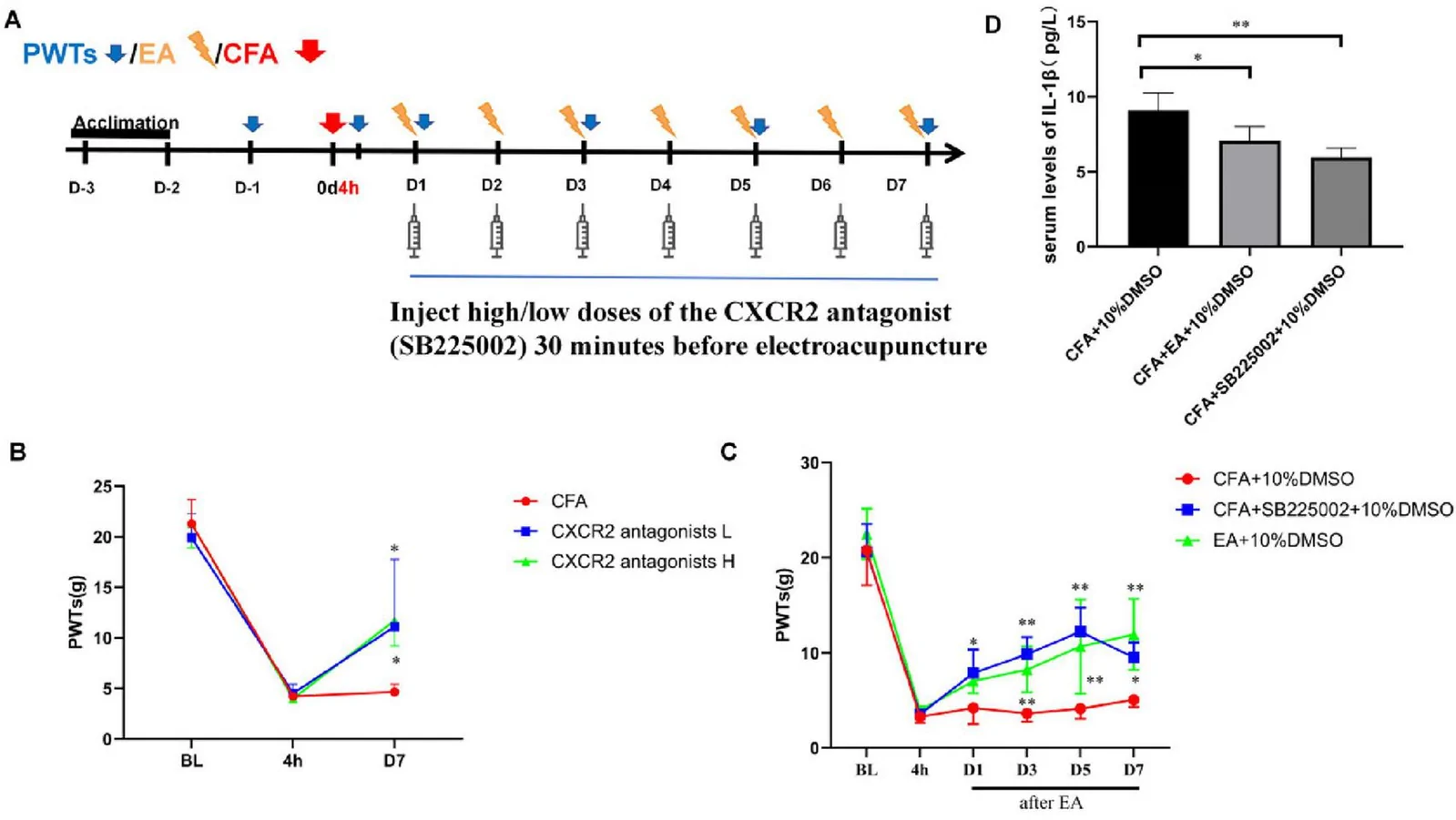
Inhibiting CXCR2 in the SCDH of rats with inflammatory pain can mimic the antinociceptive effects of EA. **(A)** Schematic representation of the experimental timeline. **(B)** The effects of different doses of CXCR2 antagonists in the spinal cord on mechanical pain in rats with inflammatory pain (*n* = 3, two-way ANOVA). **(C)** The effect of antagonizing CXCR2 receptors in the spinal cord on mechanical pain in rats with inflammatory pain (*n* = 5, two-way ANOVA). **(D)** The effect of antagonizing the CXCR2 receptor in the spinal cord on serum IL-1β levels in rats with inflammatory pain (*n* = 5, one-way ANOVA). *P < 0.05 vs. CFA+10% DMSO; **P < 0.01 vs. CFA+10% DMSO; The results are expressed as the means ± SEM; SEM, standard error of mean; EA, electroacupuncture; The higher-dose group of the CXCR2 antagonist received an intrathecal injection of a 2 μg/μl solution of SB225002; the lower-dose group of the CXCR2 antagonist received a 1 μg/μl solution; and the control group received an intrathecal injection of 10 μL of 10% DMSO solution in an equal volume. Model + SB225002 + 10% DMSO group: intrathecal injection of a 2 μg/μl SB225002 solution; Model + 10% DMSO group and EA + 10% DMSO group: intrathecal injection of 10 μL of 10% DMSO solution (equivalent volume).

To investigate the involvement of the CXCR2 receptor in the mediation of the antinociceptive effects of EA, we employed the selective CXCR2 antagonist SB225002 (Manjavachi et al., 2019; Moraes et al., 2020) to inhibit receptor activity and attenuate its function. Our findings (Figures 4A, B) indicate that intrathecal administration of SB225002 (10 μl/rat; low dose 1 μg/μl, high dose 2 μg/μl, *n* = 5) 30 min prior to daily EA significantly enhanced both the mechanical pain threshold (PWT) and the thermal pain threshold (PWL) in rats with CFA-induced inflammation. In this study, a dose of 20 μg of SB225002 was selected for intrathecal administration as the high-saturation dose. Prior experiments involving intrathecal administration have demonstrated that this dosage maximally inhibits neuroinflammation and hyperalgesia mediated by the CXCL1/CXCR2 pathway exclusively in pathological pain models (Oliveira et al., 2007; Zhou et al., 2016). Notably, the higher-dose group demonstrated a markedly significant increase in pain thresholds compared to both the CFA-induced group and the lower-dose group that receive SB225002 (*P* < 0.01). Consequently, in subsequent experiments, the higher-dose SB225002 group will be designated as the CXCR2 antagonist group to more precisely examine the efficacy and mechanism of action at this specific dosage. In comparison to the model group (Figure 4C), the mechanical pain threshold in rats subjected to EA was significantly increased on the third day post-treatment (*P* < 0.05), and demonstrated a highly significant increase by the fifth day (*P* < 0.01). This suggests that EA exerts a pronounced antinociceptive effect. Conversely, in the CXCR2 antagonist group, the mechanical pain threshold was significantly elevated on the first day following drug administration (*P* < 0.05) and showed an extremely significant increase by the third day (*P* < 0.01). Moreover, this enhancement in pain threshold exhibited a time-dependent effect, continuing to rise until the seventh day of administration. No statistically significant difference was observed between the CXCR2 antagonist group and the EA group.

Moreover, upon antagonizing CXCR2 receptor activity with SB225002, the higher-dose group exhibited a significant reduction in serum IL-1β levels (*P* < 0.01) (Figure 4D). The antinociceptive effects observed in this group did not significantly differ from those observed in the EA treatment group (*P* > 0.05). These findings suggest that the CXCR2 receptor plays a crucial role in mediating the antinociceptive effects of EA. Antagonizing the CXCR2 receptor can replicate the analgesic effects of EA in a dose-dependent manner, thereby further substantiating that the CXCL1-CXCR2 signaling axis is the principal pathway through which EA exerts its antinociceptive effects.

## 4 Discussion

Currently, a substantial body of research has established the efficacy of EA in alleviating pain. At the peripheral level, EA induces analgesia by eliciting dynamic alterations in the local tissue and extracellular microenvironment at acupoints, which subsequently modulate the activity of innervating sensory neurons either directly or indirectly (Dou et al., 2021; Fan et al., 2024). EA facilitates the local release of endogenous analgesic substances, including opioids, cannabinoids, and adenosine, which act on nociceptors to mitigate pain (Chen et al., 2009; Goldman et al., 2010). Specifically, EA activates certain ion channels, such as TRPV1 and TRPV2, at peripheral acupoints, prompting mast cells to release immune mediators like histamine and adenosine (Fan et al., 2024). Concurrently, it recruits and activates macrophages to secrete mediators such as MCP-1 and IL-6 via the NF-κB pathway. These mediators interact with receptors on nerve endings to initiate signal transduction (Hanč et al., 2023). The expression of pro-inflammatory chemokines, such as CX3CL1, is downregulated, leading to the inhibition of glial cell activation and their interaction with neurons, as well as a reduction in the release of pro-pain mediators and regulation of neurotransmitter balance, ultimately diminishing pain signal transmission (Chen et al., 2020; Gerum and Simonin, 2022). Furthermore, EA induces analgesia via central mechanisms by modulating pain-related central brain regions, neural circuits, and neuroinflammation (Wu et al., 2023; Zhang et al., 2022, 2023; Zhao, 2008). It also influences pain-associated emotional and cognitive processes, thereby alleviating negative emotions (Xu et al., 2023). Previous research has established that the CXCL1/CXCR2 signaling axis contributes to the facilitation of pathological pain within the spinal cord (Cao et al., 2014; Silva et al., 2017). This chemokine pathway enhances and propagates pain signals by promoting ERK1/2 phosphorylation, upregulating cyclooxygenase-2 (COX-2) expression, augmenting NMDAR-dependent synaptic currents, and increasing the firing frequency of excitatory postsynaptic currents (EPSCs) in dorsal horn neurons of the spinal cord (Cao et al., 2014). However, there is a notable paucity of research investigating the role of neuroinflammation mediated by the CXCL1-CXCR2 pathway in the analgesic effects of EA on inflammatory pain. This serves as confirmatory validation of a key downstream effector of electroacupuncture (EA), rather than identifying a novel mechanism. Consequently, the objective of this study is to affirm that the CXCL1/CXCR2 axis is a pivotal downstream effector pathway mediating electroacupuncture-induced analgesia.

Electroacupuncture is a therapeutic modality that integrates traditional acupuncture with electrical stimulation and has demonstrated efficacy in alleviating chronic inflammatory pain. In our study, we administered EA at a frequency of 2/100 Hz to the bilateral Zusanli (ST36) and Kunlun (BL60) acupoints in an animal model using Complete Freund’s Adjuvant (CFA). Animal studies have indicated that stimulation of the Zusanli (ST36) and Kunlun (BL60) acupoints facilitates the alleviation of inflammatory pain in rats by modulating pain-related signaling pathways (Hu et al., 2020; Wei et al., 2023). However, although the protocol of gradually increasing current (0.5–1.5 mA, +0.1 mA every 10 min) is standard for clinical translation, precludes identification of the optimal electrical dose.

Building upon the established analgesic properties of EA, we have further explored its potential molecular targets. Recent research has identified the CXCL1-CXCR2 signaling axis as a critical mediator in the initiation and progression of inflammatory pain, primarily through its role in neuroinflammation and central sensitization (Jiang et al., 2023; Lyu et al., 2021; Moraes et al., 2020). In rats, following a plantar injection of CFA, there was an upregulation of CXCL1 mRNA and protein expression in the spinal cord at 6 h and 3 days post-injection, which contributed to the development of central sensitization and inflammatory pain (Cao et al., 2014). Consequently, we investigated the expression levels of CXCL1 and CXCR2 in the dorsal horn of the spinal cord and observed a significant upregulation of both mRNA and protein levels in the dorsal horn of rats subjected to CFA-induced pain. This upregulation was closely associated with a decreased pain threshold. Notably, EA treatment effectively reversed the overexpression of CXCL1 and CXCR2. These findings preliminarily indicate that the suppression of the CXCL1–CXCR2 signaling pathway may be a crucial downstream signaling pathway responsible for the analgesic effects of EA.

Furthermore, we validated a negative correlation at the individual level between the analgesic efficacy of electroacupuncture and the abundance of spinal CXCL1/CXCR2. Rats with more substantial improvements in pain threshold exhibited greater downregulation of CXCL1/CXCR2, thereby reinforcing the mechanistic significance of this chemokine axis.

To further substantiate the involvement of the CXCL1-CXCR2 signaling axis in the analgesic process of EA, previous studies have demonstrated that intrathecal administration of recombinant CXCL1 in normal rats can induce significant nociception (Moraes et al., 2020). We utilized an intrathecal injection method to directly influence the spinal cord pathway. Initially, we administered recombinant CXCL1 at both high and low doses via intrathecal injection to exogenously elevate CXCL1 concentrations within the dorsal horn of the spinal cord. Our findings indicated that this intervention reversed the analgesic effects of EA in a dose-dependent manner, without significantly affecting serum IL-1β levels.

The complete synthetic CXCL1 precursor peptide is composed of 107 amino acids. Following the cleavage of the N-terminal signal peptide, the mature CXCL1 consists of 73 amino acids, thereby categorizing it as a high-molecular-weight peptide solute (Korbecki et al., 2022). The intact blood-spinal cord barrier only allows passive permeation of substances with molecular weights below 0.4 kDa (Bartanusz et al., 2011; Pardridge, 2005). The blood-spinal cord barrier (BSCB) is constituted by non-fenestrated endothelial cells that are interconnected through tight junction complexes (Jin et al., 2021). These intact tight junctions significantly impede the paracellular leakage of macromolecules (Bartanusz et al., 2011; Jiang et al., 2026). Consequently, as a polypeptide chemokine, CXCL1 is unlikely to traverse a structurally intact BSCB from the cerebrospinal fluid into the systemic circulation. CXCL1 is primarily synthesized and secreted by astrocytes in the spinal dorsal horn, playing a critical role in mediating central sensitization and intraspinal neuroinflammation through neuronal CXCR2 receptors (Silva et al., 2017). Concurrently, serum IL-1β is predominantly derived from macrophages infiltrating peripheral inflammatory sites in the plantar Complete Freund’s adjuvant (CFA) model (Dai et al., 2023; Xiang X. et al., 2019), rather than from central spinal cord tissue. Due to the localized spinal action of the intrathecal intervention. serum IL-1β levels remained unchanged following intrathecal CXCL1 administration.

In light of these findings and previous research, intraperitoneal administration of SB225002 in rats has been shown to significantly inhibit CFA-induced mechanical hypersensitivity, with effects persisting for up to 5 days (Manjavachi et al., 2010). Consequently, we further administered the selective CXCR2 antagonist SB225002 via intrathecal injection to inhibit the activity of CXCR2, the specific receptor for CXCL1. The findings demonstrated that SB225002 markedly elevated the pain threshold and decreased serum IL-1β levels in rats experiencing inflammatory pain. Its antinociceptive effects were comparable to those of EA. Moreover, the therapeutic efficacy of SB225002 was dose-dependent, with the higher dose group exhibiting the most pronounced improvement in pain threshold, nearing the levels observed in the sham-treated group. These findings suggest that inhibition of CXCR2 activity can replicate the antinociceptive effects of EA. and further corroborate CXCL1/CXCR2 cascade acts as a key downstream signaling pathway participating in EA-induced analgesic effect.

While CXCR2 inhibition is known to produce analgesic effects, EA, as a multi-target non-pharmacological intervention, not only suppresses spinal chemokines but also circumvents the immunosuppressive side effects typically associated with systemic CXCR2 blockade. This highlights the unique and invaluable role of EA’s holistic regulatory capabilities, representing a significant translational advantage.

## 5 Limitations

In this study, systemic inflammation was assessed by measuring only one peripheral inflammatory marker without concurrent measurement of CXCL1 in cerebrospinal fluid or other circulating chemokines and inflammatory factors. Future research involving multifactorial testing and paired cerebrospinal fluid–blood assays is necessary to further elucidate the anti-nociceptive effect of EA by regulating local signaling pathways in the spinal cord. The study primarily reports a validated association rather than a mechanistic discovery. Consequently, the upstream signaling pathways will be interrogated that regulate EA-mediated CXCL1 suppression, whether via inhibition of NF-κB phosphorylation, modulation of TLR4/MyD88 signaling, or activation of descending inhibitory pathways, which warrants further exploration in future mechanistic research. This study did not include a Sham + SB225002 group (normal rats receiving the antagonist without EA or CFA), the possibility that this antagonist exerts intrinsic analgesic effects under non-inflammatory conditions cannot be excluded. SB225002 was dissolved in 10% DMSO, and this concentration of solvent has potential neuroactive properties that may alter baseline pain thresholds. The incremental electroacupuncture (EA) current regimen is widely used in preclinical pain research for clinical translation. Furthermore, the incremental electroacupuncture (EA) current makes it difficult to identify the optimal current for reducing CXCL1/CXCR2 and achieving analgesic effects. Future studies should perform dose-response analyses using separate constant-current EA groups to overcome this issue.

## 6 Conclusion

This study elucidates that EA effectively mitigates inflammatory pain in rats by downregulating the expression of CXCL1 and CXCR2 in the SCDH and reducing serum IL-1β levels. The analgesic effect of EA is reversed upon pretreatment with recombinant CXCL1 protein, whereas intrathecal administration of the CXCR2 antagonist SB225002 replicates EA’s antinociceptive effects and significantly decreases serum IL-1β levels. Consequently, our research collectively supports the notion that the CXCL1/CXCR2 signaling axis in the SCDH as a significant downstream effector pathway contributing to EA-mediated analgesic effect. Additionally, this study provides supplements mechanistic evidence for EA analgesia.

## Data Availability

The datasets presented in this study can be found in online repositories. The names of the repository/repositories and accession number(s) can be found in the article/Supplementary material.

## Supplementary material

Supplementary Table 1 The primer sequences.

Supplementary Table 2 The Ct values of β-actin.
