# Supplementary material for: Electroacupuncture alleviates inflammatory pain by modulating the CXCL1/CXCR2 signaling axis in the spinal cord dorsal horn of rats
Source: Front Mol Neurosci. 2026 Sep 11;19:1880288. doi: 10.3389/fnmol.2026.1880288 (PMC13612514; doi:10.3389/fnmol.2026.1880288)
Supplement: Supplementary file 1 [file Table_1.docx]

Supplementary table1 The primer sequences

| Primer information | Primer names | Primer sequence（5'-3') | Length of fragment（bp） | Temperature of annealing（℃） |
| --- | --- | --- | --- | --- |
| NM_030845.2 | CXCL1-S | GTGGCAGGGATTCACTTCAAGAAC | 87 | 60 |
|  | CXCL1-A | TGTGGCTATGACTTCGGTTTGGG |  | 60 |
| NM_017183.2 | CXCR2-S | AGCAAGGAGTTCTTAGCCAAGGAG | 72 | 60 |
|  | CXCR2-A | GGTAGTGGAGGTGTTCGCTGAAG |  | 60 |
| NM_031144.3 | β-actin-S | TGTCACCAACTGGGACGATA | 165 | 60 |
|  | β-actin-A | GGGGTGTTGAAGGTCTCAAA |  | 60 |
